# HIF‐1α‐induced expression of the m6A reader YTHDF1 inhibits the ferroptosis of nucleus pulposus cells by promoting SLC7A11 translation

**DOI:** 10.1111/acel.14210

**Published:** 2024-05-23

**Authors:** Xiao Lu, Dachuan Li, Zhidi Lin, Tian Gao, Zhaoyang Gong, Yuxuan Zhang, Hongli Wang, Xinlei Xia, Feizhou Lu, Jian Song, Guangyu Xu, Jianyuan Jiang, Xiaosheng Ma, Fei Zou

**Affiliations:** ^1^ Department of Orthopedics Huashan Hospital, Fudan University Shanghai China

**Keywords:** ferroptosis, HIF‐1α, intervertebral disc degeneration, m6A, SLC7A11, YTHDF1

## Abstract

The nucleus pulposus is in a hypoxic environment in the human body, and when intervertebral disc degeneration (IVDD) occurs, the hypoxic environment is disrupted. Nucleus pulposus cell (NPC) ferroptosis is one of the causes of IVDD. N6‐methyladenosine (m6A) and its reader protein YTHDF1 regulate cellular activities by affecting RNA metabolism. However, the regulation of ferroptosis in NPCs by m6A‐modified RNAs under hypoxic conditions has not been as well studied. In this study, through in vitro and in vivo experiments, we explored the underlying mechanism of HIF‐1α and YTHDF1 in regulating ferroptosis in NPCs. The results indicated that the overexpression of HIF‐1α or YTHDF1 suppressed NPC ferroptosis; conversely, the knockdown of HIF‐1α or YTHDF1 increased ferroptosis levels in NPCs. Luciferase reporter assays and chromatin immunoprecipitation demonstrated that HIF‐1α regulated YTHDF1 transcription by directly binding to its promoter region. Polysome profiling results showed that YTHDF1 promoted the translation of SLC7A11 and consequently the expression of the anti‐ferroptosis protein GPX4 by binding to m6A‐modified SLC7A11 mRNA. In conclusion, HIF‐1α‐induced YTHDF1 expression reduces NPC ferroptosis and delays IVDD by promoting SLC7A11 translation in a m6A‐dependent manner.

## Graphical abstract

HIF‐1α regulated YTHDF1 transcription by directly binding to its promoter region. YTHDF1 promoted the translation of SLC7A11 and consequently the expression of the anti‐ferroptosis protein GPX4 by binding to m6A‐modified SLC7A11 mRNA. HIF‐1α‐induced YTHDF1 expression reduces NPC ferroptosis and delays IVDD by promoting SLC7A11 translation in a m6A‐dependent manner.

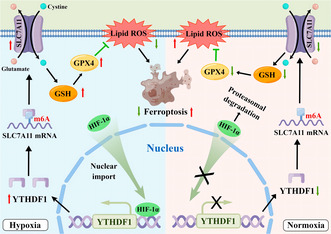

**Abbreviations**
AAV: adeno‐associated viruses
CCK‐8: cell counting kit‐8
ChIP: chromatin immunoprecipitation
CHX: cycloheximide
Co: coccyx
eIF3: eukaryotic initiation factor 3
Fer‐1: Ferrostatin‐1
GPX4: glutathione peroxidase 4
GSH: glutathione
H&E: hematoxylin & eosin
HBS: HIF‐1α‐binding sites
HIF‐1α: hypoxia inducible factor 1α
HNRNP: heterogeneous nuclear ribonucleoprotein family
IGF2BPs: insulin‐like growth factor 2 mRNA‐binding proteins
IVD: intervertebral disc
IVDD: intervertebral disc degeneration
m6A: N6‐methyladenosine
MDA: malondialdehyde
NC: negative control
NP: nucleus pulposus
NPCs: nucleus pulposus cells
OD: optical density
OE: overexpression
PRRC2A: proline‐rich coiled‐coil 2A
PUFAs: polyunsaturated fatty acid
qRT‐PCR: quantitative real time polymerase chain reaction
RIP: RNA‐binding protein immunoprecipitation assay
ROS: reactive oxygen species
shRNA: short hairpin RNA
si: small interfering
SLC7A11: solute carrier family 7 member 11
system X_c_^−^
: cysteine/glutamate transporter system
TBHP: tert‐butyl hydroperoxide
TUNEL: transferase‐mediated deoxyuridine triphosphate‐biotin Nick end labeling
WB: Western blotting
YTHDC1: YTH domain‐containing protein 1
YTHDF1: YTH N6‐methyladenosine RNA‐binding protein 1

## 1 INTRODUCTION

The intervertebral disc (IVD) is located between adjacent vertebral bodies and is an important part of the spine and is made up of endplates, the annulus fibrosus, and the nucleus pulposus (NP). Intervertebral disc degeneration (IVDD) is a very common clinical disorder. IVDD pathological changes often begin in the NP, manifesting as a decrease in the water content and aging and death of nucleus pulposus cells (NPCs) and as an imbalance in cell metabolism (Lu, Li, et al., 2022; Lu, Xu, et al., 2021; Lu, Zou, et al., 2021). As degeneration worsens, the NP protrudes from the ruptured annulus fibrosus and compresses the nerve, causing corresponding symptoms and signs. With the gradual aging of society, IVDD‐related diseases such as cervical spondylosis and lumbar disc herniation have placed an enormous burden on families and society (Molinos et al., 2023).

The IVD is the largest avascular tissue in the human body, and nutrients mainly reach disc cells from capillaries around the disc through the extracellular matrix; therefore, the NP exists in a microenvironment with no blood supply and low oxygen. Studies have shown that in hypoxic environments, hypoxia inducible factor 1α (HIF‐1α) can activate the transcription of target genes, thereby regulating processes such as angiogenesis, erythropoiesis, and glycolysis (Belozerov & Van Meir, 2005). Under hypoxic conditions, the energy metabolism of NPCs relies almost exclusively on glycolysis. HIF is crucial for maintaining the metabolic function of NPCs. HIF‐1, as an important transcription factor, participates in regulating the expression of a series of glycolysis genes and can also regulate mitochondrial energy metabolism (Guan et al., 2023; Merceron et al., 2014). HIF‐1α plays an important role in physiological activities such as extracellular matrix synthesis and energy metabolism in NPCs (He et al., 2021; Semenza, 2001; Wang et al., 2022).

The methylation of N6‐methyladenosine (m6A) is one of the most common and intensely studied types of RNA methylation; it is present through the entire RNA life cycle, plays a biological function by affecting RNA metabolism, and widely occurs in the mRNA of eukaryotic cells (Pan et al., 2021; Shen et al., 2020; Wu et al., 2023). m6A modification can affect mRNA degradation, splicing, trafficking, localization, and translation (Li et al., 2020; Liu et al., 2019). In mammalian cells, m6A modification is dynamic, reversible, and catalysed by m6A methyltransferases (METTL3, METTL14, WTAP, etc.), also known as “writers”. m6A demethylases (FTO, ALKBH5, etc.), also known as “erasers”, are responsible for removing m6A modifications from mRNA. In addition, specific RNA‐binding proteins (YTHDF1/2/3, IGF2BP1/2/3, eIF3, etc.) are referred to as “readers” and can bind to the m6A motif, affecting RNA function and producing specific phenotypic results (Li et al., 2019; Wang, Chen, Ding, et al., 2020). The results of many studies have suggested that m6A modification is widely involved in regulating IVDD development (Wang, Chen, Du, et al., 2020; Zhao et al., 2017). A recent study showed that HIF‐1α regulates YTH N6‐methyladenosine RNA‐binding protein 1 (YTHDF1), which in turn plays an important role in m6A modification (Li et al., 2021).

Ferroptosis is a regulated cell death modality discovered in recent years. With the intensive study of the mechanism of cell death, in 2012, Dixon et al. (2012) used the term ferroptosis to refer to the modality of cell death caused by iron‐dependent polyunsaturated fatty acid (PUFA) peroxide accumulation. Ferroptosis is characterized by the excessive accumulation of iron‐dependent lipid peroxides, leading to oxidative damage to the cell membrane and cell death (Li et al., 2023). Recent studies have confirmed that ferroptosis plays an important role in the pathological progression of IVDD, with ferroptosis observed in the NP, annulus fibrosus, and cartilage endplate cells (Ohnishi et al., 2022; Ru et al., 2023).

How do NPCs regulate ferroptosis in a hypoxic microenvironment? To our knowledge, there has been no related report. Further studies may provide a theoretical basis for the treatment of IVDD. In this study, we cultured NPCs in a 1% hypoxic environment to simulate the microenvironment of NPCs in vivo. We found that the overexpression of HIF‐1α, YTHDF1, and solute carrier family 7 member 11 (SLC7A11) suppressed NPC ferroptosis. HIF‐1α acts as a transcription factor for YTHDF1 to promote its expression. In addition, YTHDF1 recognizes m6A modifications on SLC7A11 mRNA and promotes translation of SLC7A11 to alleviate ferroptosis. Importantly, through in vivo experiments, we also demonstrated that the HIF‐1α/YTHDF1/SLC7A11 axis is involved in the regulation of IVDD. Collectively, the results of this study further improve the relevant mechanisms of m6A modification regulating the occurrence and development of IVDD, providing new targets for the treatment of IVDD‐related diseases.

## 2 MATERIALS AND METHODS

### 2.1 Acquisition of NP tissue and cultivation of NPCs

This study was approved by the Ethics Committee of Huashan Hospital, Fudan University (KY2022‐1039). Degenerated NP tissues were obtained from patients with cervical spondylosis, and normal NP tissues were obtained from patients with Hirayama disease (Table S1). NP tissues removed and discarded intraoperatively were collected in a 50‐mL centrifuge tube and placed on ice. Normal NP tissues were trimmed to 1‐mm^3^ pieces and subsequently placed in DMEM with 0.25% trypsin (Gibco, US) at 37°C for digestion for 30 min. The digestion medium was removed, and the remaining tissue was washed with PBS buffer (Beyotime, China) and further digested in DMEM containing 0.025% collagenase type II (Gibco, US) for 3 h at 37°C following the manufacturer's protocol. After the digestion was completed and the sample was centrifuged at 1500 R/min for 10 min, the supernatant was discarded, and the cells were resuspended in DMEM containing 10% FBS and seeded in culture flasks. The medium was changed every 3 days, and the cells were passaged until reaching a confluence of 90%. Second‐passage normal NPCs were used for subsequent experiments.

### 2.2 NPC treatments

NPCs were cultured in a 1% hypoxic environment to mimic the hypoxic microenvironment in vivo. Since normal NPCs showed low levels of ferroptosis, we treated NPCs with 100 μM tert‐butyl hydroperoxide (TBHP) for 3 h to induce ferroptosis; these NPCs were used as a control group. Depending on the subsequent grouping, cells were treated with either 5 μM erastin (a ferroptosis inducer) for 3 h, 5 μM ferrostatin‐1 (Fer‐1; a ferroptosis inhibitor) for 3 h, or 15 μmol/L LW6 (an HIF‐1α inhibitor) for 24 h (Ma et al., 2022). We overexpressed genes using plasmids and transfected NC, HIF‐1α, YTHDF1, and SLC7A11 plasmids into NPCs with LipofectamineTM3000 following the manufacturer's instructions. Small interfering (si)RNA was used to knock down genes; si‐NC, si‐HIF‐1α, si‐YTHDF1, and si‐SLC7A11 were purchased from Shanghai GenePharma Co., Ltd.

### 2.3 Western blotting (WB)

NPCs were gently washed three times with PBS buffer (AS1025, ASPEN), and 100 μL of RIPA protein lysis solution (AS1004, ASPEN) was added to each well. The cells were incubated on ice for 30 min, followed by centrifugation at 12,000 R/min for 30 min. The supernatant was retained, and the protein concentration was determined with a BCA protein concentration assay kit (AS1086, ASPEN) following the manufacturer's instructions. Protein loading buffer (AS1011, ASPEN) was added to protein samples, which were then mixed and boiled in a water bath for 5 min. Gels were poured following the instruction provided with an SDS–PAGE kit (AS1012, ASPEN), and proteins were electrophoresed at a constant current (100 mA) for 80 min and then transferred to a membrane at a constant voltage (180 V) for 2 h. PVDF membranes (IPVH00010, Millipore) were blocked in 5% nonfat milk for 1 h and incubated overnight at 4°C with primary antibodies against HIF‐1α, YTHDF1, SLC7A11, GPX4, and GAPDH. After washing three times with TBST, the membrane was incubated with secondary antibodies for 1 h at room temperature, and the protein bands were subsequently visualized with an ECL luminescent reagent (AS1059, ASPEN) and an imaging system. Protein expression was calculated as the grey value for the protein band of interest divided by the grey value for GAPDH (internal reference) band.

### 2.4 Immunohistochemical staining

Specimens were fixed in 10% neutral formalin and embedded in paraffin. Sections were deparaffinized, hydrated, rinsed in PBS, incubated in 3% hydrogen peroxide solution to block endogenous peroxidase, fixed at high temperature and pressure for 2.5 min, blocked in goat serum, incubated with primary antibodies at 37°C for 2 h, returned to room temperature, rinsed in PBS, incubated with secondary antibodies for 20 min at room temperature, rinsed in PBS, developed with DAB (ZLI‐9019, ZSBIO), counterstained in haematoxylin (H9627‐25G, Sigma) after rinsing, and mounted for visualization under a light microscope.

### 2.5 Cell counting Kit‐8 (CCK‐8) assay

NPCs were seeded in 96‐well plates at 104/well in a volume of 100 μL/well, and 10 μL of CCK‐8 assay solution (G021‐1‐1, Nanjing Jiancheng Bio) was added to each well. After 2.5 h of incubation, the optical density (OD) of each well at 450 nm was measured with a microplate reader. We calculated the results using a previously reported method (Lu, Xu, Lin, Zou, et al., 2023).

### 2.6 Measurement of Fe2+ and malondialdehyde (MDA) levels

The Fe^2+^ content and MDA level were measured using an iron assay kit (BC4355, Solarbio) and a cellular MDA assay kit (A003‐4‐1, Nanjing Jiancheng Bio), respectively. The specific procedures were performed according to the kit instructions.

### 2.7 Measurement of intracellular reactive oxygen species (ROS) levels by flow cytometry

Following the kit instructions, DCFH‐DA (S0033, Beyotime) was added to resuspend the NPCs, which were then incubated for 20 min at 37°C inside a cell incubator and mixed by inversion every 3 min to ensure adequate contact between the probe and the cells. Under an excitation wavelength of 480 nm and emission wavelength of 535 nm, ROS levels were measured using flow cytometry (CytoFLEX, Beckman).

### 2.8 MitoSOX red staining for mitochondrial ROS detection

MitoSOX red reagent (M36008, Thermo) was added to cells after they were rinsed with PBS. After incubation in the dark at 37°C for 30 min, the nuclei were stained with Hoechst 33342 solution (B8040, Solarbio) and then observed and photographed under a fluorescence microscope (Eclipse Ci‐L, Nikon).

### 2.9 Quantitative real‐time polymerase chain reaction (qRT–PCR)

Total RNA was extracted from cells with the TRIzol reagent (EP013, ELK Biotechnology) and reverse transcribed into cDNA (EQ031, ELK Biotechnology). qRT–PCR was performed to detect HIF‐1α, YTHDF1, SLC7A11, and GPX4 mRNA expression using SYBR Green PCR kits (EQ001, ELK Biotechnology). The primer sequences are shown in Table S2. Relative gene expression was calculated using the 2^−ΔΔCt^ method.

### 2.10 Dual‐luciferase reporter assay

Luciferase‐associated plasmids were constructed by Shanghai GENECHEM Company. The corresponding gene promoters or control plasmids were cotransfected with HIF‐1α plasmids into NPCs, and luciferase activity was detected using a dual‐luciferase reporter assay kit (ab228530, Abcam).

### 2.11 Chromatin immunoprecipitation (ChIP)‐PCR

Experiments were performed according to the ChIP kit instructions (Millipore, Germany). After crosslinking with formaldehyde, NPCs were disrupted with an ultrasonic disruptor. An anti‐HIF‐1α antibody was added to the cells, followed by mixing overnight at 4°C with rotation. The next day, the antibody‐bound protein–DNA complexes were collected by adding protein G magnetic beads and then eluted, and protein–DNA crosslinks were reversed. The DNA was obtained after treatment and purification with proteinase K. DNA templates were used for real‐time PCR to measure DNA that immunoprecipitated with the antibodies of interest.

### 2.12 Online tools

JASPAR was used to predict transcription factor‐binding sites (http://jaspar.genereg.net). SRAMP was used to predict m6A modification sites on mRNA (http://www.cuilab.cn/sramp). Graphical abstract was drawn by Figraw (https://www.figdraw.com).

### 2.13 RNA‐binding protein immunoprecipitation assay (RIP)

RIP lysate (Millipore, Germany) was added to NPCs, and after lysis on ice for 30 min, the supernatant was collected by centrifugation at 12,000 rpm for 10 min. After mixing the lysates, antibodies were added, and the samples were incubated overnight at 4°C. The next day, Protein A/G magnetic beads (Millipore, Germany) prepared with RIP buffer were added to the lysis solution, and the samples were incubated at 4°C for 4 h. After ensuring quality by Western blotting, the remaining lysate was used to extract RNA with TRIzol reagent to perform qRT–PCR.

### 2.14 RNA and protein stability assay

NPCs were treated with 5 μg/mL actinomycin D (Millipore, Germany) for 0, 3, 6, and 9 h, and the relative levels of SLC7A11 mRNA were determined by qRT–PCR at the corresponding time points. Additionally, NPCs were treated with 100 μg/mL cycloheximide (CHX; Millipore, Germany) for 0, 3, 6, and 9 h, and the relative levels of SLC7A11 protein were detected by Western blotting at the corresponding time points. RNA and protein half‐lives were calculated according to previous studies (Li et al., 2021; Shen et al., 2020).

### 2.15 Polysome profiling

NPCs were treated with CHX (100 μg/mL) for 10 min at 37°C. The cells were then lysed on ice using lysis buffer. Finally, following the instructions provided with Gradient Station (BioComp Instruments, Fredericton, Canada), RNA was extracted and subjected to qRT–PCR analysis.

### 2.16 Establishment of a rat model of IVDD

This study was approved by the Animal Ethics Committee of Fudan University (202210011S). The overexpression of genes in animal experiments was achieved by the transfection of adeno‐associated viruses (AAVs; Hanbio Biotechnology Shanghai Co., Ltd.) (Wu et al., 2022). Gene knockdown was achieved by the transfection of AAV containing short hairpin RNA (shRNA; Shanghai OBiO Technology) (Dai et al., 2022). Fifty SD rats aged 12 weeks were selected and divided into the following five groups using the random number table method: acupuncture (IVDD) group, AAV‐NC group, AAV‐YTHDF1 group, sh‐NC group, and sh‐SLC7A11 group (*n* = 10 each group). Rats were anaesthetized by the intraperitoneal injection of 2% pentobarbital sodium 50 mg/kg. The Co7/8 and Co8/9 vertebral bodies were located by palpation, and a 20 G needle was perpendicularly inserted just above the disc into the centre of the nucleus pulposus and rotated 360°; the needle remained in place for 30 s, and the depth of puncture was approximately 5 mm (Lu, Xu, Lin, Song, et al., 2023). After modeling, corresponding interventions were immediately performed. The IVDD group was intradiscally injected with 4 μL of saline. The AAV‐NC group was intradiscally injected with 2 μL of saline+2 μL of AAV‐NC. The AAV‐YTHDF1 group was intradiscally injected with 2 μL of saline+2 μL of AAV‐YTHDF1. The sh‐NC group was intradiscally injected with 2 μL of sh‐NC + 2 μL of AAV‐YTHDF1. The sh‐SLC7A11 group was intradiscally injected with 2 μL of sh‐SLC7A11 + 2 μL of AAV‐YTHDF1.

### 2.17 Transferase‐mediated deoxyuridine triphosphate‐biotin nick end labeling (TUNEL) fluorescence staining

TUNEL staining was performed with prepared sections (as described above) following the product manual provided with the reagent kit (G1504, Servicebio). After the sections were fixed and permeabilized, the reaction mixture was prepared according to the instructions and added dropwise following the appropriate steps. After staining and sealing, the sections were viewed under a fluorescence microscope. At high magnification, six fields of view were selected from each slice to count TUNEL‐positive (green) cells and cell nuclei (DAPI, blue).

### 2.18 Statistical analysis

The experimental data were processed by GraphPad Prism 9.5.0 software (GraphPad Software, La Jolla, CA, USA), and the data are expressed as the mean ± SD. When the normal distribution was satisfied (Shapiro–Wilk W test) and the variance was homogeneous, the data between the two groups were compared by *t*‐test, the data between the multiple groups were compared by single factor analysis of variance (ANOVA). When the data does not follow the normal distribution or the variance is uneven, the data between the two groups were compared by Wilcoxon rank‐sum test, the data between the multiple groups were compared by Kruskal‐Wallis test. The LSD test was used for pairwise comparison. The bilateral inspection level was *α* = 0.05.

## 3 RESULTS

### 3.1 HIF‐1α alleviated ferroptosis in NPCs

**FIGURE 1:**
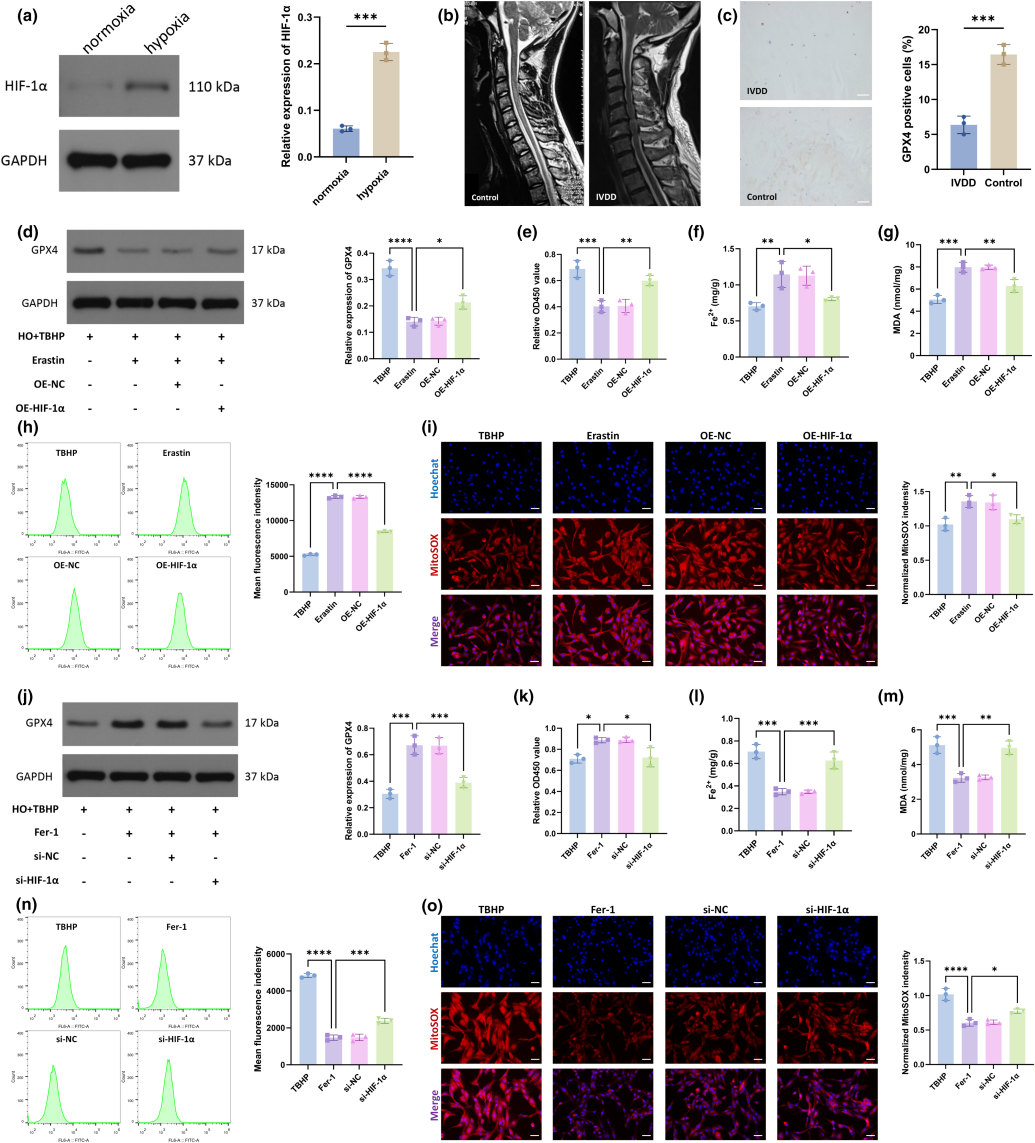
HIF‐1α alleviated ferroptosis in NPCs. (a) WB was used to measure HIF‐1α expression in the NPCs under normoxia and hypoxia environments. (b) MRI of patients with Hirayama disease (control group) and cervical spondylosis (IVDD group). (c) Immunohistochemistry was used to detect the expression of GPX4 in the NP tissues of the control group and the ICDD group. Scale bar: 200 μm. (d) WB was used to detect GPX4 expression in the NPCs. HO: hypoxia. (e) CCK‐8 kit was used to detect cell viability. (f) Detecting the relative levels of Fe2+ in NPCs. (g) Detecting the relative levels of MDA in NPCs. (h) Total intracellular ROS levels were detected by flow cytometry. (i) MitoSOX staining was used to detect intramitochondrial ROS levels. The intensity of red fluorescence represents the level of ROS. Scale bar: 20 μm. (j) WB was used to detect GPX4 expression in the NPCs. (k) CCK‐8 kit was used to detect cell viability. (l) Detecting the relative levels of Fe2+ in NPCs. (m) Detecting the relative levels of MDA in NPCs. (n) Total intracellular ROS levels were detected by flow cytometry. (o) MitoSOX staining was used to detect intramitochondrial ROS levels. Scale bar: 20 μm. (TBHP group: HO + TBHP group. Erastin group: HO + TBHP+Erastin group. OE‐NC group: HO + TBHP+Erastin+OE‐NC group. OE‐HIF‐1α group: HO + TBHP+Erastin+OE‐HIF‐1α group. Fer‐1 group: HO + TBHP+Fer‐1 group. si‐NC group: HO + TBHP+Fer‐1 + si‐NC group. si‐HIF‐1α group: HO + TBHP+Fer‐1 + si‐HIF‐1α group. *n* = 3. Data are presented as the mean ± SD of three independent experiments. *p < 0.05, **p < 0.01, ***p < 0.001, ****p < 0.0001, ns: not significant).

HIF‐1α is an important protein produced by cells to adapt to hypoxic environments. In this study, we cultured NPCs under 1% hypoxia (HO) to mimic the hypoxic microenvironment in which the NP resides in vivo (He et al., 2021; Lu, Xu, Lin, Song, et al., 2023). Consistent with previous studies, NPCs highly expressed HIF‐1α protein in the hypoxic environment (Figure 1a). We collected NP tissue from the control and IVDD groups. For the control group, the NP was obtained from postoperative tissue of patients with Hirayama disease, and the NP from these patients showed a high signal on MRI without degeneration (Lu, Xu, et al., 2021; Lu, Zou, et al., 2021; Lu, Zou, et al., 2022). For the IVDD group, the NP was obtained from patients with cervical spondylosis, and the NP from these patients showed a low signal on MRI, indicating degeneration of the NP (Figure 1b, Table S1). Glutathione peroxidase 4 (GPX4) is a key protein that inhibits ferroptosis (Li et al., 2023). Immunohistochemistry suggested a significant decrease in GPX4 content in the NP of the IVDD group compared with that in the NP of the control group (Figure 1c). We induced ferroptosis with tert‐butyl hydroperoxide (TBHP) in vitro to construct a cellular model to study ferroptosis in NPCs. Erastin, a ferroptosis inducer, caused a decrease in GPX4 levels in NPCs, and the overexpression of HIF‐1α led to an increase in GPX4 levels (Figure 1d). The results of CCK‐8 assays suggested that erastin significantly reduced the viability of NPCs and that the overexpression of HIF‐1α reversed this effect (Figure 1e). The intracellular Fe^2+^ level can reflect the level of ferroptosis, and the overexpression of HIF‐1α reduced the cellular Fe^2+^ overload caused by erastin (Figure 1f). The change in malondialdehyde (MDA) content represents the level of lipid peroxidation and indirectly reflects the attack by free radicals on cells. The results showed that erastin increased MDA levels in NPCs; that trend was not observed after the overexpression of HIF‐1α (Figure 1g). Correspondingly, erastin increased the total intracellular reactive oxygen species (ROS) (Figure 1h) and mitochondrial ROS content (Figure 1i), but the overexpression of HIF‐1α partially cleared the accumulated ROS in the cells.

In addition, we alleviated TBHP‐induced ferroptosis through ferrostatin‐1 (Fer‐1), a ferroptosis inhibitor, and then knocked down HIF‐1α with small interfering siRNA to further investigate the role of HIF‐1α in ferroptosis. Fer‐1 increased the GPX4 expression level in and viability of NPCs and reduced the accumulation of Fe^2+^ caused by TBHP; however, these effects were attenuated when HIF‐1α was inhibited (Figure 1j–l). In addition, the levels of intracellular total ROS, mitochondrial ROS, and lipid peroxides decreased after treatment with Fer‐1, but this effect was not observed when HIF‐1α was inhibited (Figure 1m–o). Overall, the above results suggested that HIF‐1α alleviated ferroptosis in NPCs.

### 3.2 HIF‐1α is a transcription factor for YTHDF1

**FIGURE 2:**
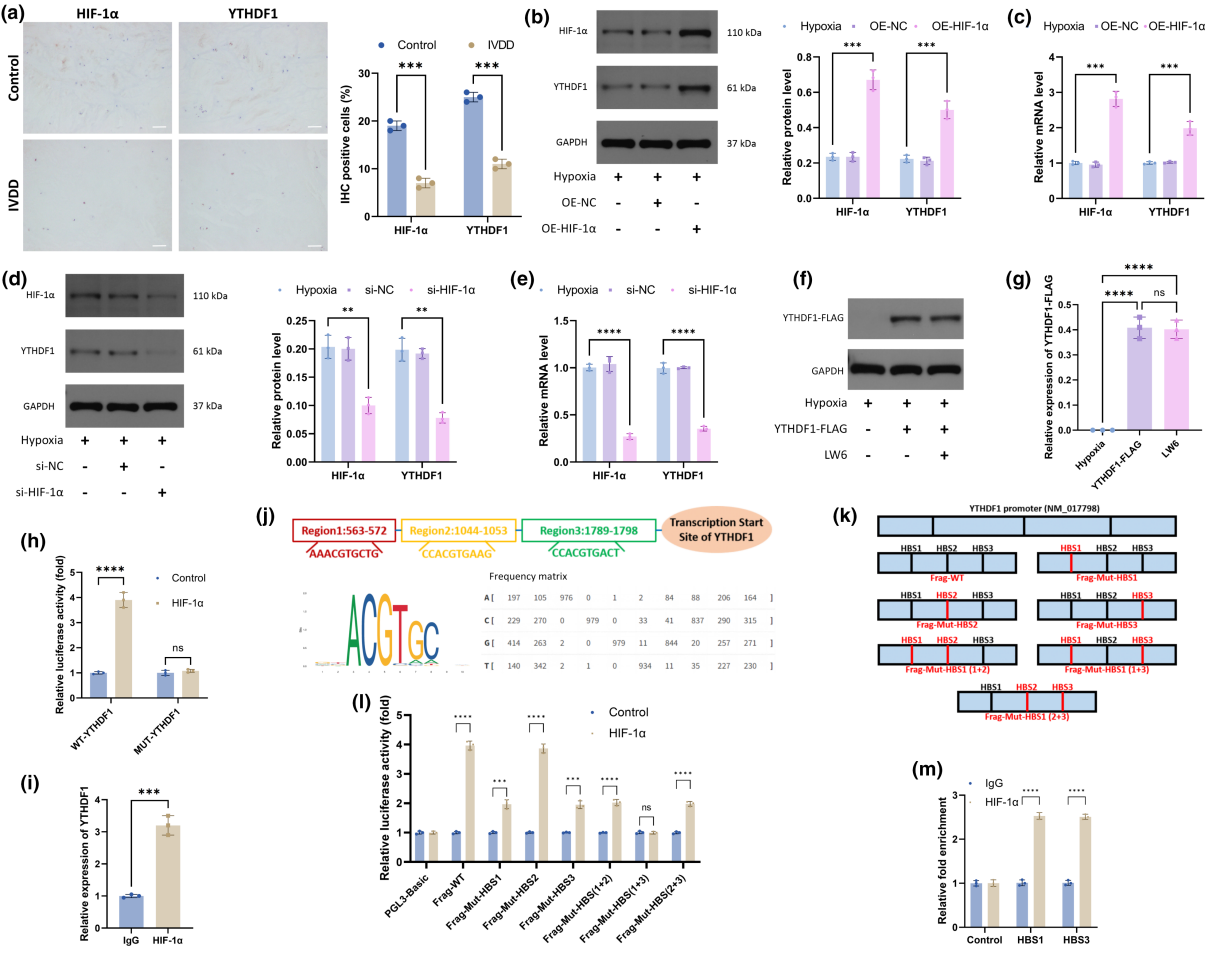
HIF‐1α is a transcription factor for YTHDF1. (a) Immunohistochemistry was used to detect HIF‐1α and YTHDF1 in the NP of control and IVDD groups. (b) WB was used to determine the protein levels of HIF‐1α and YTHDF1. (c) qRT‐PCR was used to determine the mRNA levels of HIF‐1α and YTHDF1. (d) WB was used to determine the protein levels of HIF‐1α and YTHDF1. (e) qRT‐PCR was used to determine the mRNA levels of HIF‐1α and YTHDF1. (f) WB was used to examine the levels of YTHDF1 in in CMV‐driven FLAG‐YTHDF1‐overexpressing NPCs treated with or without LW6. (g) Semiquantitative analysis of YTHDF1‐FLAG. (h) Dual luciferase reporter was used to verify the interaction between HIF‐1α and YTHDF1. (i) ChIP‐qPCR assays confirmed HIF‐1α occupancy at YTHDF1 promoters in NPCs. (j) The online tool Jaspar was employed to identify putative HIF‐1α‐binding sites (HBS) in the genomic sequence adjacent to the transcription start site of the YTHDF1 gene. (k) Mutations were made sequentially for each HBS. (l) Dual luciferase reporter was used to detect the interaction between HIF‐1α and MUT‐YTHDF1. (m) ChIP‐qPCR was used to detect the binding between HIF‐1α and HBS. (*n* = 3. Data are presented as the mean ± SD of three independent experiments. **p < 0.01, ***p < 0.001, ****p < 0.0001, ns: not significant).

We next investigated the relationship between HIF‐1α and YTHDF1. The expression levels of HIF‐1α and YTHDF1 were higher in the NP of the control group than in the NP of the IVDD group (Figure 2a). After the overexpression of HIF‐1α, YTHDF1 significantly increased at both the protein and RNA levels (Figure 2b,c).

Similarly, when HIF‐1α was inhibited, the expression level of YTHDF1 also decreased substantially (Figure 2d,e). In addition, the use of the HIF‐1α inhibitor LW6 after transfection with YTHDF1‐FLAG did not affect the expression of YTHDF1‐FLAG (Figure 2f,g). These results indicated a positive correlation between HIF‐1α and YTHDF1 and that the regulation of YTHDF1 by HIF‐1α occurred before translation.

Luciferase reporter analysis showed that HIF‐1α interacted with WT‐YTHDF1 and promoted its transcription but did not interact with MUT‐YTHDF1 (Figure 2h). Chromatin immunoprecipitation qPCR (ChIP–qPCR) assays confirmed HIF‐1α occupancy at YTHDF1 promoters in NPCs (Figure 2i). The online tool Jaspar was employed to identify putative HIF‐1α‐binding sites (HBSs) in the genomic sequence adjacent to the transcription start site of the YTHDF1 gene. We observed three putative HBSs within the genomic region (NM_017798) (Figure 2j). We next mutated each HBS and examined luciferase activity (Figure 2k). When HBS1 or HBS3 was mutated, luciferase activity decreased by approximately half. When both were mutated simultaneously, luciferase activity was abolished (Figure 2l). ChIP–qPCR analysis showed that chromatin fragments containing HBS1 or HBS3 were more readily enriched by the anti‐HIF‐1α antibody (Figure 2m). Collectively, the above results demonstrated that HIF‐1α is a transcription factor for YTHDF1.

### 3.3 YTHDF1 alleviated ferroptosis in NPCs

**FIGURE 3:**
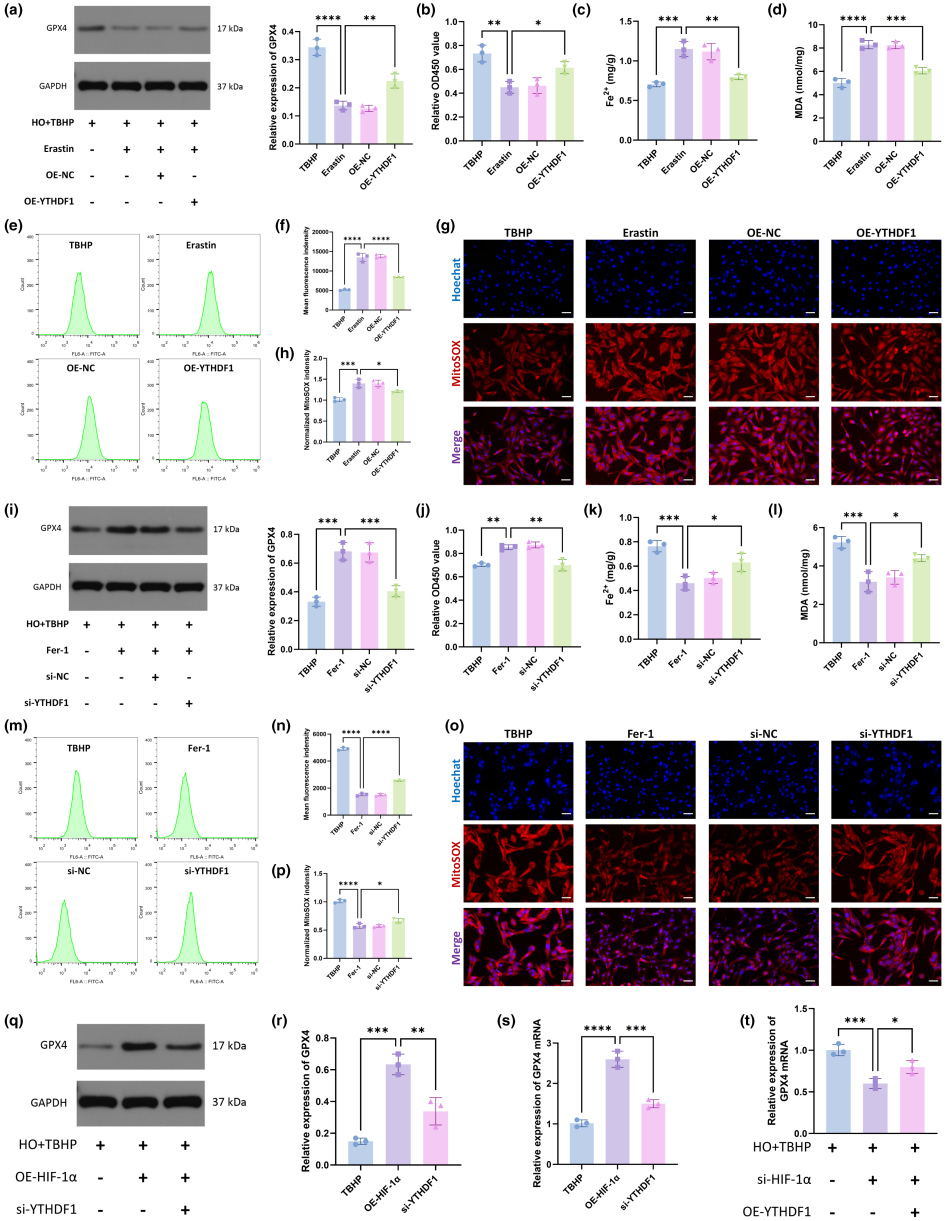
YTHDF1 alleviated ferroptosis in NPCs. (a) WB was used to detect GPX4 expression in the NPCs. (b) CCK‐8 kit was used to detect cell viability. (c) Detecting the relative levels of Fe2+ in NPCs. (d) Detecting the relative levels of MDA in NPCs. (e) Total intracellular ROS levels were detected by flow cytometry. (f) Quantification analysis of ROS mean fluorescence intensity. (g) MitoSOX staining was used to detect intramitochondrial ROS levels. Scale bar: 20 μm. (h) Relative fluorescence intensity of MitoSOX. (i) WB was used to detect GPX4 expression in the NPCs. (j) CCK‐8 kit was used to detect cell viability. (k) Detecting the relative levels of Fe2+ in NPCs. (l) Detecting the relative levels of MDA in NPCs. (m) Total intracellular ROS levels were detected by flow cytometry. (n) Quantification analysis of ROS mean fluorescence intensity. (o) MitoSOX staining was used to detect intramitochondrial ROS levels. Scale bar: 20 μm. (p) Relative fluorescence intensity of MitoSOX. (TBHP group: HO + TBHP group. Erastin group: HO + TBHP+Erastin group. OE‐NC group: HO + TBHP+Erastin+OE‐NC group. OE‐YTHDF1 group: HO + TBHP+Erastin+YTHDF1 group. Fer‐1 group: HO + TBHP+Fer‐1 group. si‐NC group: HO + TBHP+Fer‐1 + si‐NC group. si‐YTHDF1 group: HO + TBHP+Fer‐1 + si‐YTHDF1 group.) (q) WB was used to detect GPX4 expression in the NPCs. (r) Semiquantitative analysis of GPX4 protein. (s) qRT‐PCR was used to determine the mRNA levels of GPX4. (t) qRT‐PCR was used to determine the mRNA levels of GPX4. (*n* = 3. Data are presented as the mean ± SD of three independent experiments. *p < 0.05, **p < 0.01, ***p < 0.001, ****p < 0.0001, ns: not significant).

We next investigated the role of YTHDF1 in ferroptosis in NPCs. We first overexpressed YTHDF1 to observe whether it can alleviate ferroptosis caused by erastin. From the results, the overexpression of YTHDF1 reversed the erastin‐induced decrease in GPX4 levels and cell viability and the erastin‐induced increase in Fe^2+^ (Figure 3a–c). In addition, the overexpression of YTHDF1 mitigated erastin‐induced oxide accumulation, including MDA (Figure 3d), total intracellular ROS (Figure 3e,f), and mitochondrial ROS (Figure 3g,h).

We next knocked down YTHDF1. si‐YTHDF1 impaired the inhibitory effect of Fer‐1 on ferroptosis. Specifically, after knocking down YTHDF1, the expression of GPX4 and cell viability decreased (Figure 3i,j), and intracellular Fe^2+^ (Figure 3k), MDA (Figure 3l), total intracellular ROS (Figure 3m,n), and mitochondrial ROS (Figure 3o,p) levels increased. The above results indicated that YTHDF1 alleviated ferroptosis in NPCs. Furthermore, we found that the attenuation of ferroptosis by the overexpression of HIF‐1α was abolished after the inhibition of YTHDF1 (Figure 3q–s). In contrast, the promotion of ferroptosis by si‐HIF‐1α was weakened when YTHDF1 was overexpressed (Figure 3t). These findings suggested that HIF‐1α may exert an inhibitory effect on ferroptosis through YTHDF1.

### 3.4 The function of YTHDF1 in attenuating ferroptosis is dependent on m6A‐binding pockets in the YTH domain

**FIGURE 4:**
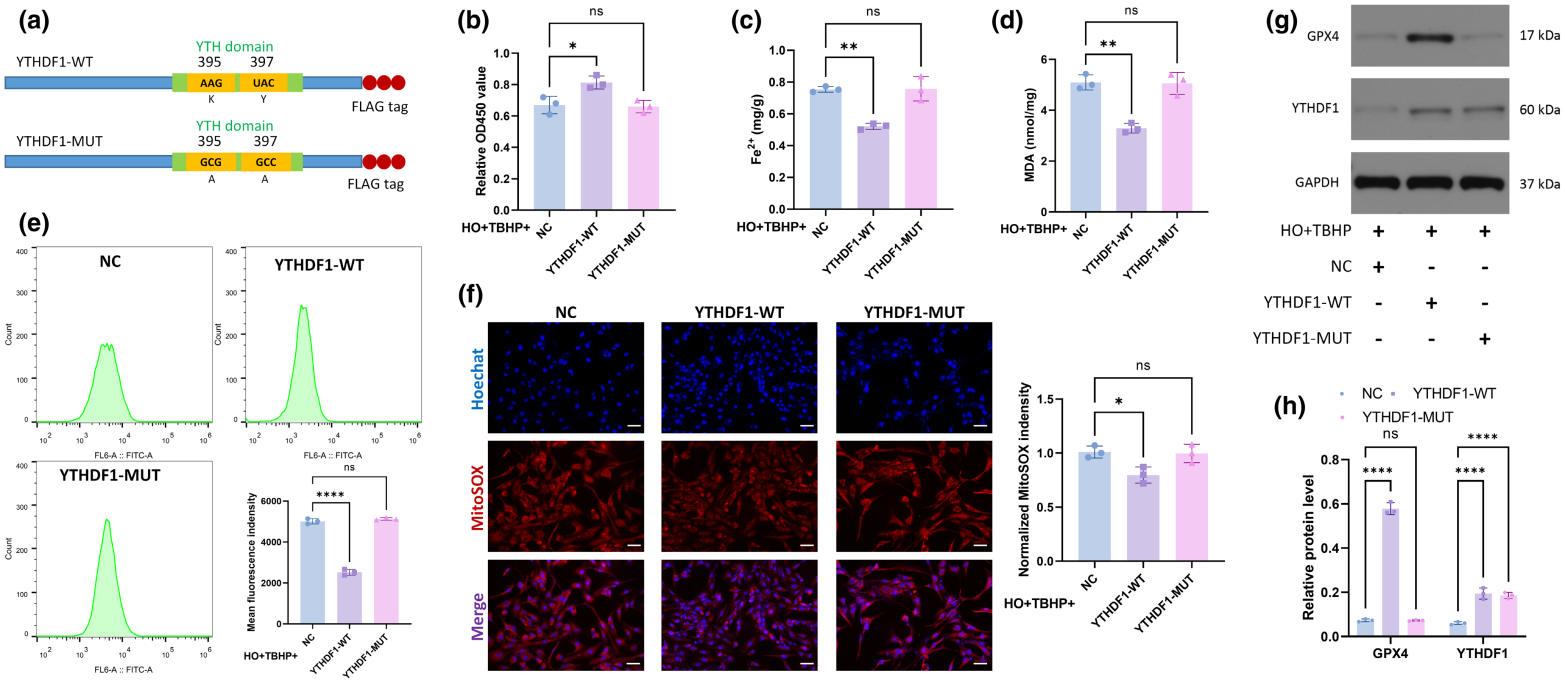
The function of YTHDF1 in attenuating ferroptosis is dependent on m6A‐binding pockets in the YTH domain. (a) YTHDF1's ability to recognize m6A modifications depends on m6A‐binding pockets in the YTH domain. (b) CCK‐8 kit was used to detect cell viability. (c) Detecting the relative levels of Fe2+ in NPCs. (d) Detecting the relative levels of MDA in NPCs. (e) Total intracellular ROS levels were detected by flow cytometry. (f) MitoSOX staining was used to detect intramitochondrial ROS levels. Scale bar: 20 μm. (g) WB was used to detect GPX4 expression in the NPCs. (h) Semiquantitative analysis of GPX4 and YTHDF1 protein. (*n* = 3. Data are presented as the mean ± SD of three independent experiments. *p < 0.05, **p < 0.01, ****p < 0.0001, ns: not significant).

As an m6A “reader”, the ability of YTHDF1 to recognize m6A modifications depends on m6A‐binding pockets in the YTH domain. Mutations in K395 and Y397 abrogated the capacity of YTHDF1 to bind with mRNA (Li et al., 2021) (Figure 4a). Therefore, we mutated the YTH domain to determine whether YTHDF1 exerts an inhibitory effect on ferroptosis by recognizing m6A modifications. As expected, only WT‐YTHDF1 increased cell activity and reduced Fe^2+^ (Figure 4b,c). In addition, WT‐YTHDF1 attenuated TBHP‐induced intracellular peroxide accumulation (Figure 4d–f) and promoted GPX4 expression (Figure 4g,h). MUT‐YTHDF1 did not have the ability to rescue NPCs. Thus, we can conclude that the function of YTHDF1 in attenuating ferroptosis is dependent on m6A‐binding pockets in the YTH domain.

### 3.5 YTHDF1 promotes the translation of SLC7A11 mRNA upon binding to it

**FIGURE 5:**
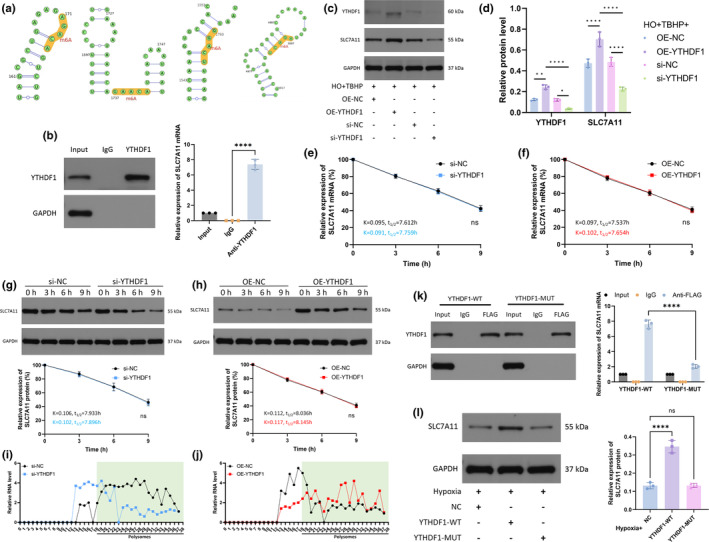
YTHDF1 promotes translation of SLC7A11 mRNA upon binding to it. (a) SRAMP online tool was employed to predict the m6A modification sites of SLC7A11 mRNA. (b) RIP‐derived protein and RNA in NPCs examined using Western blotting and RT‐qPCR, respectively. (c) WB was used to detect YTHDF1 and SLC7A11 expression in the NPCs. (d) Semiquantitative analysis of YTHDF1 and SLC7A11 protein. (e) After treating NPCs with actinomycin D and si‐YTHDF1, mRNA was detected by qRT‐PCR at 0, 3, 6, and 9 h, respectively. The Y‐axis represents the relative level of mRNA compared to 0 h. (f) After treating NPCs with actinomycin D and OE‐YTHDF1, mRNA was detected by qRT‐PCR at 0, 3, 6 and 9 h, respectively. (g) After treating NPCs with CHX and si‐YTHDF1, protein was detected by WB at 0, 3, 6, and 9 h, respectively. The Y‐axis represents the relative level of protein compared to 0 h. (h) After treating NPCs with CHX and OE‐YTHDF1, protein was detected by WB at 0, 3, 6, and 9 h, respectively. (i) After treating NPCs with si‐YTHDF1, qRT‐PCR analysis of SLC7A11 mRNA distribution in different ribosome populations. (j) After treating NPCs with OE‐YTHDF1, qRT‐PCR analysis of SLC7A11 mRNA distribution in different ribosome populations. (k) RIP‐derived protein and RNA in NPCs examined using western blotting and RT‐qPCR, respectively. (l) WB was used to detect SLC7A11 expression in the NPCs. (*n* = 3. Data are presented as the mean ± SD of three independent experiments. *p < 0.05, **p < 0.01, ****p < 0.0001, ns: not significant).

The SRAMP online tool was employed to predict the m6A modification sites of SLC7A11 mRNA. The results showed that there were four sites with moderate predictive strength or higher (Figure 5a). RIP suggested that YTHDF1 could bind to SLC7A11 mRNA (Figure 5b). The expression of SLC7A11 increased after the overexpression of YTHDF1, and the two were positively correlated (Figure 5c,d). These results implied that YTHDF1 promoted SLC7A11 protein expression after binding to SLC7A11 mRNA.

We further treated NPCs with actinomycin D (a transcription inhibitor) and found that after knocking down YTHDF1, the SLC7A11 mRNA level decreased to the same extent at various time points as SLC7A11 mRNA in control cells (Figure 5e). In addition, the overexpression of YTHDF1 did not affect the decrease in the SLC7A11 mRNA level (Figure 5f). These results indicated that YTHDF1 did not affect the stability of SLC7A11 mRNA. Additionally, we treated cells with the protein translation inhibitor cycloheximide (CHX) and found that YTHDF1 did not affect the stability of the SLC7A11 protein, as it did not affect the degradation rate of the SLC7A11 protein (Figure 5g,h). However, polysome profiling demonstrated that YTHDF1 knockdown resulted in a decrease in SLC7A11 mRNA in the translation fractions (Figure 5i). In contrast, the overexpression of YTHDF1 increased SLC7A11 mRNA in the translation fractions (Figure 5j). These results suggested that YTHDF1 regulated the translation of SLC7A11.

RIP analysis showed that WT‐YTHDF1 effectively immunoprecipitated SLC7A11 mRNA in NPCs but that the interaction between MUT‐YTHDF1 and SLC7A11 mRNA was significantly weakened, indicating that the m6A‐binding pockets of YTHDF1 were crucial for its binding to SLC7A11 mRNA (Figure 5k). Furthermore, Western blotting showed that WT‐YTHDF1, rather than MUT‐YTHDF1, promoted SLC7A11 expression (Figure 5l).

### 3.6 YTHDF1 mitigates ferroptosis in NPCs through the regulation of SLC7A11

**FIGURE 6:**
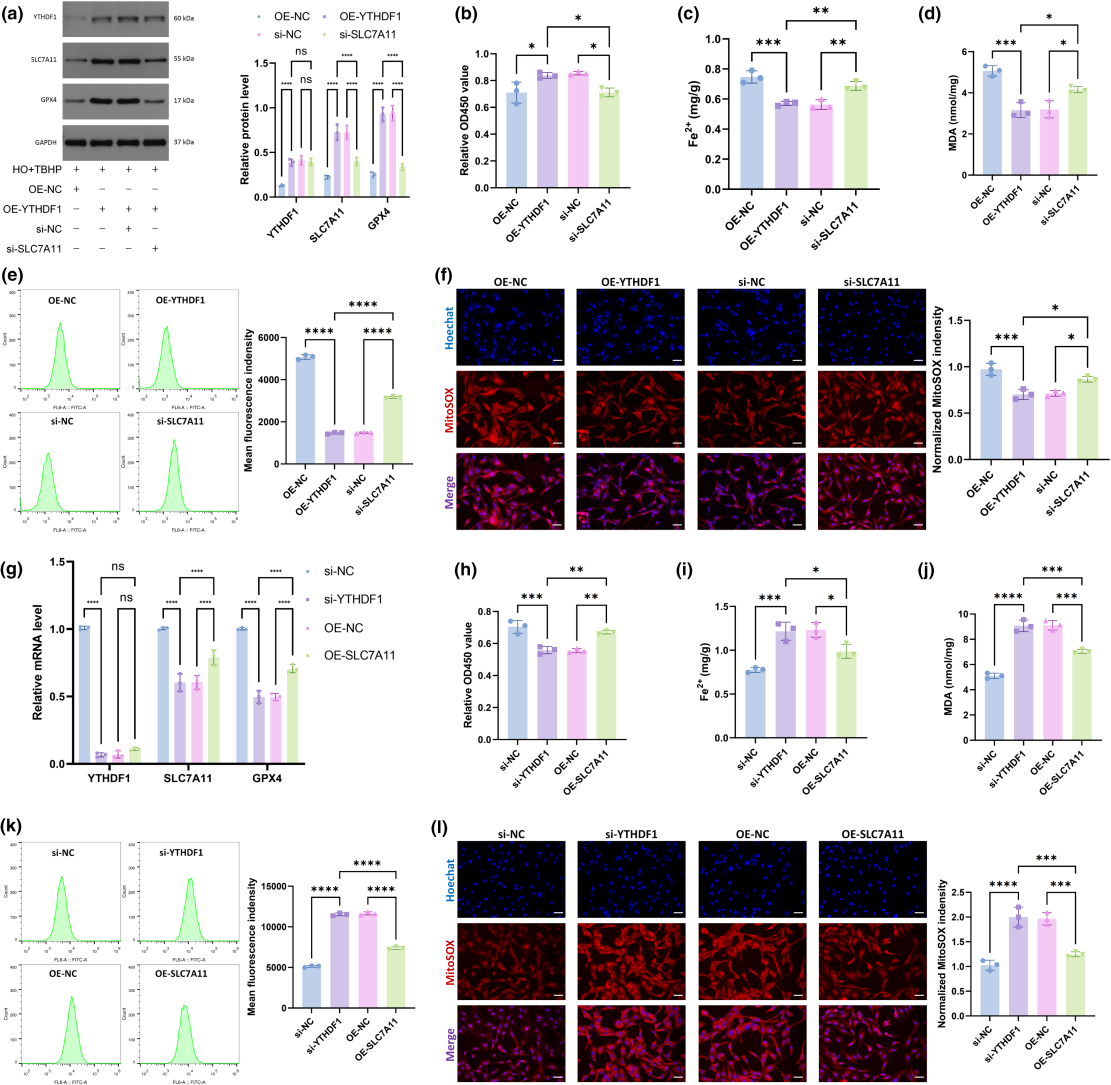
YTHDF1 mitigates ferroptosis in NPCs through regulation of SLC7A11. (a) WB was used to detect YTHDF1, SLC7A11, and GPX4 expression in the NPCs. (b) CCK‐8 kit was used to detect cell viability. (c) Detecting the relative levels of Fe2+ in NPCs. (d) Detecting the relative levels of MDA in NPCs. (e) Total intracellular ROS levels were detected by flow cytometry. (f) MitoSOX staining was used to detect intramitochondrial ROS levels. Scale bar: 20 μm. (OE‐NC group: HO + TBHP+OE‐NC group. OE‐YTHDF1 group: HO + TBHP+OE‐YTHDF1 group. si‐NC group: HO + TBHP+OE‐YTHDF1 + si‐NC group. si‐SLC7A11 group: HO + TBHP+OE‐YTHDF1 + si‐SLC7A11 group.) (g) qRT‐PCR was used to detect YTHDF1, SLC7A11, and GPX4 mRNA expression levels in the NPCs. (h) CCK‐8 kit was used to detect cell viability. (i) Detecting the relative levels of Fe2+ in NPCs. (j) Detecting the relative levels of MDA in NPCs. (k) Total intracellular ROS levels were detected by flow cytometry. (l) MitoSOX staining was used to detect intramitochondrial ROS levels. Scale bar: 20 μm. (si‐NC group: HO + TBHP+si‐NC group. si‐YTHDF1 group: HO + TBHP+si‐YTHDF1 group. OE‐NC group: HO + TBHP+si‐YTHDF1 + OE‐NC group. OE‐SLC7A11 group: HO + TBHP+si‐YTHDF1 + OE‐SLC7A11 group.) (*n* = 3. Data are presented as the mean ± SD of three independent experiments. *p < 0.05, **p < 0.01, ***p < 0.001, ****p < 0.0001, ns: not significant).

When we knocked down SLC7A11, the expression of YTHDF1 was not affected, and the expression of GPX4 decreased (Figure 6a). Similarly, when SLC7A11 was inhibited, the effect of the overexpression of YTHDF1 on easing ferroptosis became limited (Figure 6b–f). We further suppressed YTHDF1 and overexpressed SLC7A11 and found that the overexpression of SLC7A11 had no effect on YTHDF1 expression but increased the level of GPX4 mRNA (Figure 6g). Furthermore, the overexpression of SLC7A11 rescued the aggravation of NPC ferroptosis caused by the inhibition of YTHDF1 (Figure 6h–l). The above results suggested that YTHDF1 played a role in reducing ferroptosis through the upstream regulation of SLC7A11.

### 3.7 YTHDF1 alleviates ferroptosis in vivo and delays IVDD in rats

We further investigated the role of YTHDF1 in vivo. Acupuncture of the intervertebral disc causes degeneration of the caudal intervertebral disc in rats. Subsequently, we injected adeno‐associated virus (AAV) with YTHDF1 into the IVDs of rats to overexpress YTHDF1 and injected AAV with short hairpin (sh)RNA to knock down SLC7A11. Consistent with the results of the in vitro experiments, YTHDF1 increased the expression levels of SLC7A11 and GPX4 in vivo (Figure S1A–D). In addition, acupuncture caused the degeneration of the IVD, and Fe^2+^ and MDA levels increased, indicating that ferroptosis occurred. AAV‐YTHDF1 alleviated ferroptosis in intervertebral discs; however, this therapeutic effect was significantly attenuated after SLC7A11 was knocked down (Figure S1E,F). TUNEL staining suggested that AAV‐YTHDF1 alleviated the death of NPCs caused by acupuncture but that sh‐SLC7A11 increased the death rate (Figure S1G,H). These results suggest that YTHDF1 delayed IVDD in vivo.

## 4 DISCUSSION

The IVD is a special structure without blood vessels and is in an ischaemic and hypoxic microenvironment under normal physiological conditions. The homeostatic balance of the IVD microenvironment is the basis for maintaining the normal function of IVDs. Members of the HIF family play important roles in maintaining the homeostatic balance of the IVD microenvironment. The HIF family includes several members, among which HIF‐1α is widely expressed in various cells and is considered a master regulator of metabolism, the cell cycle, angiogenesis, and tumorigenesis (Silagi et al., 2021). Studies have shown that during the occurrence and development of IVDD, HIF‐1α is involved in various aspects, such as extracellular matrix metabolism, the inflammatory response, apoptosis, and the regeneration and repair of IVDs (He et al., 2021; Silagi et al., 2021). Richardson et al. (2008) found that HIF‐1α promotes glycolysis by regulating glucose transporters in hypoxic environments. Ha et al. (2006) found that the expression of HIF‐1α in degenerative IVD tissue was associated with the level of NPC apoptosis. Therefore, the expression of HIF‐1α is related to the stability of the microenvironment in which IVD tissue resides.

However, in the late stage of IVDD, neovascularization occurs, which increases the oxygen content in the NP (Chacko et al., 2010; Nerlich et al., 2007), thereby accelerating the oxygen‐dependent degradation of HIF‐1α (Jiang et al., 1997). Therefore, in this study, we focused on HIF‐1α degradation during IVDD as the initiating factor of ferroptosis in NPCs by combining the characteristics of the hypoxic microenvironment of the NP. The immunohistochemistry results showed that HIF‐1α expression was higher in normal NP tissue than in degenerative NP tissue and that HIF‐1α was induced under hypoxic conditions. The overexpression of HIF‐1α reversed the decrease in GPX4 caused by erastin and alleviated ferroptosis in NPCs. In contrast, knocking down HIF‐1α exacerbated ferroptosis in NPCs (Figure 1). In addition, HIF‐1α, as a transcription factor of YTHDF1, participated in the regulation of m6A modification (Figure 2).

m6A refers to the methylation of the sixth nitrogen atom of adenine. m6A modifications mainly occur at the stop codon, the 3′ noncoding region, and the RRACH sequence of exons (Dominissini et al., 2012). The m6A modification process is conserved, dynamic, and reversible and is dependent on a variety of related proteins and enzymes, including methyltransferases (writers), demethylases (erasers), and methylation‐related specific binding proteins (readers). The m6A modification of RNA participates in various cellular biological processes by regulating RNA metabolism, mRNA stability, and gene expression, playing a crucial role in mammalian development and disease progression. Zhao et al. (2017) found that m6A‐modified miR‐129‐5p can promote autophagy in NPCs, leading to IVDD. Additionally, there have been reports that m6A‐modified lncRNA LOC102555094 is also involved in regulating the development of IVDD (Wang, Chen, Du, et al., 2020).

m6A‐binding proteins play roles in regulating RNA metabolism, processing, translation, and stability by recognizing m6A methylation sites. The m6A‐binding proteins mainly include YTH domain family proteins, heterogeneous nuclear ribonucleoproteins (HNRNPs), insulin‐like growth factor 2 mRNA‐binding proteins (IGF2BPs), eukaryotic initiation factor 3 (eIF3), and proline‐rich coiled‐coil 2A (PRRC2A) (Li et al., 2021; Li et al., 2019; Wang, Chen, Ding, et al., 2020). The YTH domain family proteins include YTH N6‐methyladenosine RNA‐binding protein 1 (YTHDF1), YTH N6‐methyladenosine RNA‐binding protein 2 (YTHDF2), YTH N6‐methyladenosine RNA‐binding protein 3 (YTHDF3), YTH domain‐containing protein 1 (YTHDC1), and YTH domain‐containing protein 2 (YTHDC2) (X. Wang et al., 2014). YTHDF2 was the first m6A‐binding protein discovered and can promote mRNA degradation and reduce the stability of targeted transcripts (Du et al., 2016). YTHDF1 can enhance mRNA translation and promote protein synthesis. YTHDF3 is interconnected with YTHDF1‐2 and plays an important role in regulating the specific binding of YTHDF1‐2 to RNA. Functionally, YTHDF3 can interact with YTHDF1 to enhance the translation of methylated mRNA, promote protein expression, and enhance YTHDF2‐mediated mRNA degradation and instability (A. Li et al., 2017). YTHDF1‐3 are involved in the regulation of mRNA metabolism, processing, translation, and degradation by m6A methylation in an integrated and cooperative manner.

In this study, we found that YTHDF1 recognizes the m6A modification site of SLC7A11 through its m6A‐binding pocket in the YTH domain, thereby promoting the translation of SLC7A11 and reducing ferroptosis in NPCs (Figures 3, 4, 5).

Ferroptosis, although classified as regulated cell death, can be differentiated from other types of regulated cell death by morphological and biochemical characteristics (D. Li et al., 2023). The main morphological features of ferroptosis include reduced mitochondrial volume, blurred cristae, outer membrane rupture, increased membrane density, and an intact nuclear membrane. Biochemical features include inhibition of the cysteine/glutamate transporter system (system X_c_^−^), decreased glutathione (GSH) synthesis, decreased GPX4 activity, and the accumulation of iron ions and PUFA peroxides. GPX4, as the main endogenous antioxidant enzyme, can convert toxic lipid peroxides into nontoxic lipid alcohols and is considered a core inhibitor of ferroptosis (Ohnishi et al., 2022). However, the system X_c_^−^‐GSH‐GPX4 pathway must function properly. System X_c_^−^ consists of a light chain, SLC7A11, and a heavy chain SLC3A2, and its function is to pump out glutamic acid and import cystine. Cystine is subsequently reduced to cysteine and is involved in the synthesis of GSH. Ultimately, assisted by GSH, GPX4 changes peroxyl bonds generated by lipid peroxidation to hydroxyl bonds, and lipid peroxides lose their peroxidative activity.

To our knowledge, we are the first to report that in the hypoxic microenvironment, NPCs regulate YTHDF1 through HIF‐1α, thereby regulating the system X_c_^−^‐GSH‐GPX4 pathway, reducing ferroptosis, and delaying IVDD.

There are some limitations to the present study. First, HIF‐1α may also regulate other “readers”, and we only investigated YTHDF1 in this study. Second, in the study of m6A modifications, we focused on “readers” and ignored “writers” and “erasers”. Second, in the study of ferroptosis, we focused on the system X_c_^−^‐GSH‐GPX4 pathway without considering other signaling pathways. We will conduct further research on these issues in the future. Finally, this study did not focus on the morphological and functional changes of mitochondria in ferroptosis, which will be investigated in future studies.

Collectively, we found that HIF‐1α is a transcription factor for YTHDF1 and can promote the expression of YTHDF1. YTHDF1 recognizes the m6A modification site of SLC7A11 through its m6A‐binding pocket in the YTH domain, thereby promoting the translation of SLC7A11 and reducing NPC ferroptosis. During the process of IVDD, HIF‐1α undergoes oxygen‐dependent degradation, and the level of ferroptosis in the NP increases (Figure S2). For the first time, we revealed the underlying mechanism by which the NP maintains physiological functions in a hypoxic environment. This study may provide a theoretical basis for the treatment of IVDD and provide a new target for drug development.

## AUTHOR CONTRIBUTIONS

XL, DL, ZL, and TG: Methodology, Software, Writing—original draft. ZG and YZ: Methodology, Software. HW, XX, FL, and JS: Supervision; Validation; Visualization. GX, JJ, XM, and FZ: Funding acquisition, Investigation, Project administration. All authors have read and approved the final manuscript.

## CONFLICT OF INTEREST STATEMENT

The authors declare that they have no conflict of interest.

## Supporting information


Appendix S1.

## Data Availability

The data that support the findings of this study are available in the main text or the Supplementary Materials or from the corresponding author upon reasonable request.
