# Supplementary material for: HIF‐1α‐induced expression of the m6A reader YTHDF1 inhibits the ferroptosis of nucleus pulposus cells by promoting SLC7A11 translation
Source: Aging Cell. 2024 May 23;23(9):e14210. doi: 10.1111/acel.14210 (PMC11488328; doi:10.1111/acel.14210)
Supplement: Supplementary file 1 — Appendix S1. [file ACEL-23-e14210-s001.pdf]

Supplemental Table 1. Demographic data of intervertebral disc donors

Control group (Hirayama disease group)				
No.	Age	Sex	Disc level	Pfirrmann grade
1	16	Male	C4-5, C5-6	1
2	16	Male	C4-5, C5-6	2
3	18	Male	C4-5, C5-6	1
4	17	Female	C4-5, C5-6	2
5	17	Male	C4-5, C5-6	2

IVDD group (Cervical spondylosis group)				
No.	Age	Sex	Disc level	Pfirrmann grade
1	59	Female	C4-5, C5-6	4
2	61	Female	C4-5, C5-6	4
3	64	Female	C4-5, C5-6	4
4	65	Male	C4-5, C5-6	5
5	63	Male	C4-5, C5-6	4

Supplemental Table 1. The demographic data including age, sex, operative disc levels, and Pfirrmann grade for disc tissue samples obtained at surgery.

Supplemental Table 2. The primer sequences

Primers	Base sequence (5’ -3’)	
H-GAPDH	sense	CATCATCCCTGCCTCTACTGG
	antisense	GTGGGTGTCGCTGTTGAAGTC
H-HIF-1α	sense	TGATTGCATCTCCATCTCCTACC
	antisense	GTAGTTCTTTGACTCAAAGCGACAG
H-YTHDF1	sense	ATAGCAACTCTCCTGGAAACGTC
	antisense	GATGGAGTACTTAATGGAGCGG
H-SLC7A11	sense	TGTGGGGTCCTGTCACTATTTG
	antisense	GATATCACAGCAGTAGCTGCAGG
H-GPX4	sense	AGGAGCCAGGGAGTAACGAA
	antisense	AGCCGTTCTTGTCGATGAGG

Supplemental Figure 1

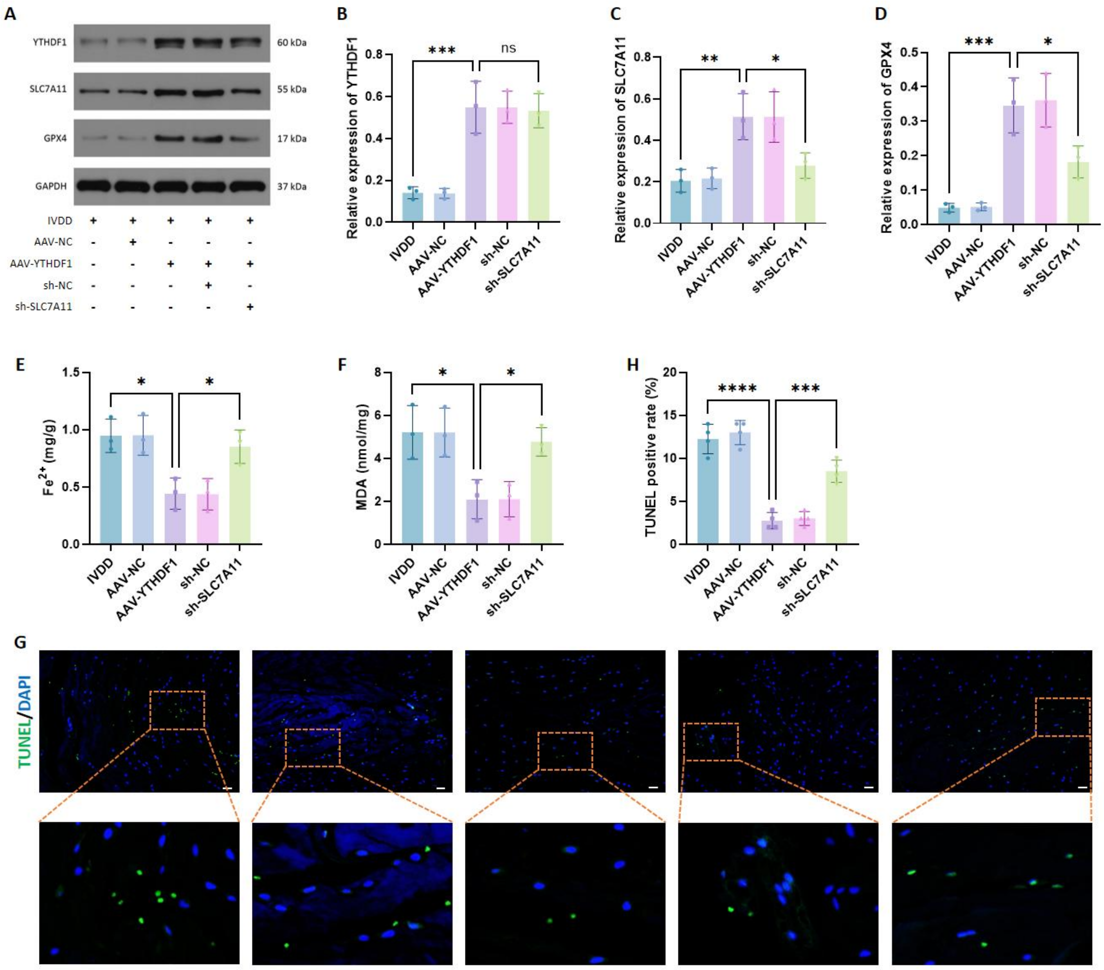

Supplemental Figure 1. YTHDF1 alleviates ferroptosis in vivo and delays IVDD in rats. (A-D) WB was used to detect YTHDF1, SLC7A11, and GPX4 expression in the NP tissues. And conduct semi-quantitative analysis of the results. (E) Detecting the relative levels of Fe2+ in NP tissues. (F) Detecting the relative levels of MDA in NP tissues. (n = 3. Data are presented as the mean ± SD of three independent experiments.) (G) TUNEL staining of nucleus pulposus was used to detect cell death. Scale bar: 50 μm. (H) Quantitative analysis of TUNEL staining. (n = 4. Data are presented as the mean ± SD of ten independent experiments. *P < 0.05, **P < 0.01, ***P< 0.001, ****P< 0.0001) (IVDD group: Acupuncture group without any intervention. AAV-NC group: IVDD+AAV-NC group. AAV-YTHDF1 group: IVDD+AAV-YTHDF1 group. Sh-NC group: IVDD+AAV-YTHDF1+sh-NC group. Sh-SLC7A11 group: IVDD+AAV-YTHDF1+sh-SLC7A11 group.)

Supplemental Figure 2

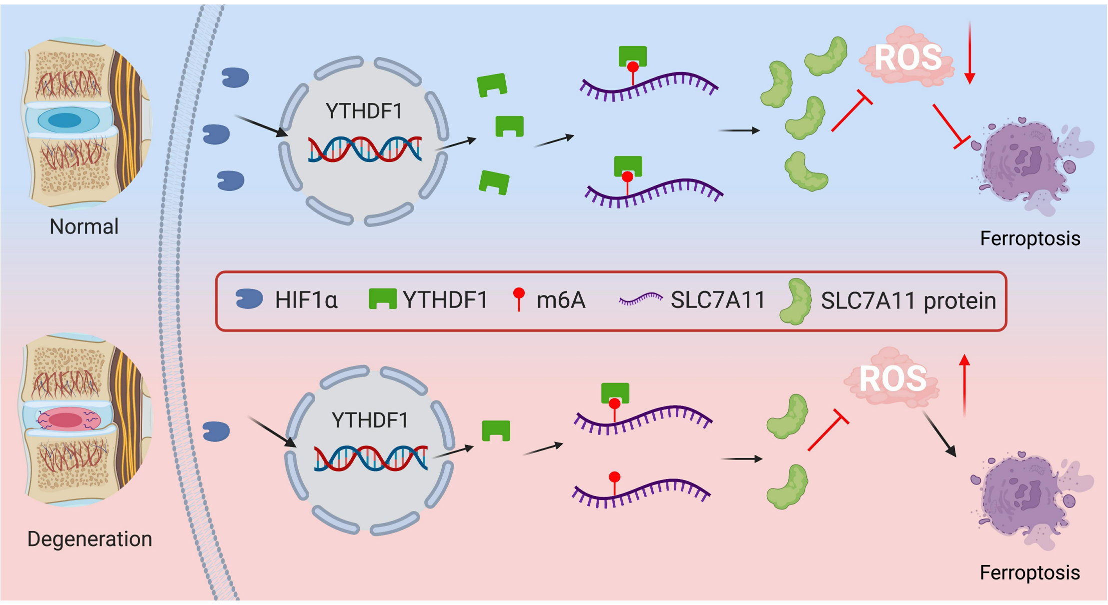

Supplemental Figure 2. The main findings of this study. HIF-1α-induced expression of the m6A reader YTHDF1 inhibits the ferroptosis of nucleus pulposus cells by promoting SLC7A11 translation.
